# Medical News and Miscellany

**Published:** 1889-04

**Authors:** 


### Medical News and Miscellany.

"Has a Homeopath—(known as such,) the right to use other than homeopathic means in the treatment of a case?"

This question was propouuded to Judge Geo. C. Barrett, Supreme Court of New York, who decided in the negative. He says:

"If I call in a medical man who designates himself a "homoeopathic physician" it is because I don't wish to be treated allopathically or electrically or otherwise than homeopathically." Common honesty demands that before a confiding patient (in hands of a declared homeopath) is to be drugged with quinine, iron, morphine or other medicaments, he should be told that the homeopathic system has failed, and that relief can be had only by a change of system; the homeopath is in honor bound—according to Judge Barrett's decision, to "give small doses of a single drug—administered upon the principle of "*similia, similibus, curentur*;"—if he do otherwise, he is a fraud and a cheat.

This position has been held and proclaimed by the Journal *ab initio*.

More Doctors Walker!—It runs in the family! We are much gratified to notice the announcement that Drs. E. R. and W. H. Walker, sons of our esteemed friend and correspondent Dr. W. W. Walker of Schulenburg, have graduated with distinction at Tulane University Medical Department, and have returned home, where they received an ovation at the hands of their friends.

Revision of the U. S. Pharmacopœia.—A call has been issued for a general convention to assemble in Washington, D. C., at noon May 7, 1890, for the purpose of providing for a revision and publication of the Pharmacopœia of the United States. Every incorporated medical or pharmacal College, Association or Society desiring to be represented in the convention, is requested to send to Dr. Robert Armory, Boston Mass., its corporate title and a list of its officers. It should be addressed to the care of Dr. Edwin H. Brigham, Assistant Librarian of the Boston Medical Library, 19 Boylston Place, Boston,—in order that Dr. Armory may prepare for publication, as directed by the convention of 1880—a list of the bodies to be represented.

---

MARRIED in Webberville, Texas, March 21, ult. Dr. L. D. Hill to Miss Kate Taylor, both of that city. Congratulations of the JOURNAL.

---

WORKS BOTH WAYS.—Customer (to boy in drug store). "Have you anything for the removal of superfluous hair?"

Boy: "Yes sir; there's a bottle of our celebrated Hair Elixir. One dollar, please."

Customer: "But that's intended to make the hair grow."

Boy (whispering): "I know that's what it says on the label, but you take my word and try a bottle."

---

Physician: "Patrick, don't you know better than to have your pig-pen so close to the house?

Patrick: "An' phy should Oi not, sor?"

Physician: "It's unhealthy."

Patrick: "Be away wid yer nonsense! Sure the pig has never been sick a day in his life."

---

"Now, doctor," he said, as he joined the medical gentleman in the street, "in the case of a man who can't sleep at night what would you advise?"

"I would advise him to sleep in the day-time."

---

BIOGRAPHY OF CONTEMPORARY PHYSICIANS OF TEXAS.—This work so auspiciously begun will be pushed to completion within the present year. It was suspended in consequence of the extra ordinary stringency in money, produced by two years of drougth: And again, we made a contract with S. H. Dixon to finish the canvas for subscriptions and he was unable to do so, partly for the above reasons and partly because his associate who was to furnish the necessary funds disappointed him; hence he was obliged to throw up the contract. We take pleasure in announcing that arrangements are now being made for a throrough canvass by an experienced man, who will have a solid financial backing, and as fast as subscribers are taken their biographies will be prepared for the press, and it is hoped to issue the work in good style by fall. F. E. DANIEL.

---

INFORMATION WANTED of one "Doctor E. G. Jones", late 272 Union Square, Patterson, N. J. We have been carrying his advertisement for six months on his order, and now we cannot find him by letter or sight draft; the latter is returned unpaid, and endorsed by the Patterson Bank, "no attention paid to our notices". As many of our contemporaries are running the said advertisement, perhaps some of them can give us the information desired, and inform us what success they have had in the way of collecting their bills.

---

TO THE MEMBERS TEXAS STATE MEDICAL ASSOCIATION.—This JOURNAL publishes all the official documents and announcements of the State Association, *con amore.* It does so for the benefit and information of members, and *no where else* can certain information be found. We are at pains to learn all the news in the medical profession and give it to our readers. In advance of each meeting there is found in our columns all the details of said meeting that members desire to know. For this reason alone, it would be to the interest of members to subscribe for the JOURNAL, outside of any consideration of moral duty to aid a laudable effort to foster and build up medical organization all over the State, and to advance medical science in every department.

A copy of this number is sent complimentary to each member of the Association, not a subscriber, in order that he may see certain details of the approaching meeting in which all are interested. Subscription solicited.

---

DR. M. L. HAGGARD, of Mountain Peak, whose health was much shattered in the sand flats of his former home, has entirely regained his health, and is doing a large and laborious practice. This will be good news to his large circle of friends all over Texas—he being one of the most popular as well as "pushing" members of the State Association.

---

WE HAVE RECEIVED from Dr. Jno. H. Rauch, Secretary Illinois State Board of Health, a "Report on the Water Supply of Illinois and the Pollution of its Rivers," which we will review at leisure.

Also, a neat little pamphlet in morocco, from Messrs. W. R. Warner & Co., entitled "Warner's Reference Handbook of Therapeutics," containing much valuable information. It is one of the best "ready made prescription" books we have seen.

---

ONCE MORE!—We are mortified and made ashamed to say that only about two-thirds of the members of the *Physicians' Mutual Benefit Association* have paid assessments No. 9, 10 and 11. Brothers Buck, Jameson and Maddox, faithfully paid every call on them, and now that their families, *who really need it*, are expecting the small amount due on assessments for *their* benefit, one third of the members "go back" on their *written pledge*, to "promptly pay one dollar for the benefit of a deceased brother's family, when notified so to do, by the Secretary." We *must* believe it is inadvertence, and not intentional neglect. Shame, shame! Every physician who has a tender wife, or a bright eyed little curly headed darling should *join* this Association and *cheerfully* give *a dollar* to the orphans of a departed brother! We make this one more appeal before closing the list, whereby all who have not paid, would be cut off, and give them one more chance to redeem a written pledge, and perform a sacred duty.

---

DR. R. H. EANES, of Taylor, Texas, passed through Austin on the 15th inst. en route to New Orleans to take a special course at the Polyclinic.

---

REMOVALS.—Dr. B. F. King, of Burnett, has removed to Lampasas, and Dr. T. N. Skeen, from Winsboro to Wichita Falls. Dr. J. S. Letcher, of Lampasas, to Dallas. Dr. Gibson, a recent graduate of Bellevue, has located at Austin; Dr. Hamilton, of Austin, recently graduated at Louisville, has also located at Austin.

---

THE AUSTIN DISTRICT MEDICAL SOCIETY held its sixth quarterly meeting in Austin, March 21, ult. There were present thirty physicians. Two new members were admitted, Dr. W. H. Way, recently settled in Austin, and Dr. J. S. Watson, of Burnett. A resolution was adopted, instructing the delegates to the T. S. M. A., to memorialize said association, to apply for a charter at once. The society is in a flourishing condition. The papers read at the meeting, will hereafter appear in the JOURNAL.

---

FOR CHRONIC CHILLS, and that peculiar but very common condition of chronic malarial blood poisoning seen in swampy and other malarial sections, in which there is a "fever cake" or enlarged spleen, and a general dropsical tendency, there is no remedy, or combination of remedies, which has a better effect than that known throughout the South as Gadberry's Spleen Mixture. It is a solution of the oxy-sulphate of iron and potassa, and is made as follows:

| | | |
|---|---|---|
| ℞ | Pulv. Ferri Sulph | ʒi. |
| | Acid Nitrici | ʒi. |

Mix, and when reaction has ceased, add one ounce of some aromatic water, mint or cinnamon; to this add quinine ʒi, little by little, stirring constantly.

| | | |
|---|---|---|
| ℞ | Potas. Citratis vel Nitratis | ʒi. |
| | Aguæ Menth vel Cin | ℥vii. |

Mix and dissolve and add slowly to the above, stirring constantly. Filter and wrap in blue or other dark paper, to exclude the light.

When properly made the mixture should be a perfectly clear, transparent green fluid. The dose for an adult is a tablespoonful, three times a day, and in cases of long standing, it should be given every day for a month. On the days on which the chill is expected, the mixture should be given in anticipation, the same as quinine is given, and for children the dose should be proportioned to the age.

If old, dried sulphate of iron be used, the mixture is apt to be brown, and to deposit a sediment. If the sulphate be used in the natural state as found in the shops, and it is preferable, the mixture will be a bright green, and clear.

Care is necessary in adding the quinine to the acid solution of iron, to prevent its lumping; if added gradually and stirred with a grass rod it will dissolve like snow.

---

TO LIMIT MARRIAGE.—A bill, (*Albany Medical Annals*) has been introduced in the legislature of Kentucky, which prohibits marriage with an idiot, lunatic, pauper, vagrant, tramp, gambler, felon, or any person rendered physicaily or mentally helpless or unfit for the marriage relation, or any person *with a violent tempe1*.

---

PUT DOWN YOUR WORK DOCTOR for a few days, and come to San Antonio to the meeting; come with a determination to add something to the interest of the occasion and with a heart attuned to harmany and brotherly love, help along the car of progress; help to build up a State Association of which your children's children will be proud. Let no recollection of past discords; let no feeling of jealousy or resentment, or prejudice find place in your heart; but come for a general fraternal greeting and interchange of friendly courtesies. There will be a grand banquet and we can "down our sorrows," you know.

---

INGROWING NAILS are a great affliction. Various remedies have been proposed and tried, but in our experience nothing equals the application of carbolic acid. Brush the acid lightly over the inflamed surface, and allow it to penetrate under the nail. In 24 hours it will be found that the nail is partly softened, and can be removed without pain, while the acid has acted as a complete anæsthetic to the tender inflamed tissue. Applied in the same way to a sprain or a bruise, in which the skin is not broken, it affords instant relief. To corns also, after paring, it is a great relief,—provided, it is not put on too thick, so as to blister.

---

FOR PSORIASIS.—

| | | |
|---|---|---|
| ℞ | Acid Chrysophanici | ʒss |
| | Traumaticine | ℥i. |

M.

S. Apply with a camel hair pencil. —*Daniel.*

---

FOR BARBER'S ITCH.—

| | |
|---|---|
| Hydrarg Ammoniat | grs. x |
| Bismuth Subnit. | ʒi |
| Liq. Carbonis Deterg | ʒi |
| Lanolin | . M. |

Liq. apply night and morning. —*Exchange.*

---

RINGWORM.—A saturated solution of salicylic acid in collodion is said to be a prompt cure for ringworm. One application generally suffices.

---

SEBORRHŒA SICCA, (Dry Scales on Scalp.) Friction with Hebras lotion, equal parts green soap and alcohol, applied with a soft sponge, will remove the crust; after which an inunction with any emolient ointment will relieve the trouble, oxide of zinc ointment is good.

---

SEBORRHOEA SICCA, another good application as used by Vida[1] is,

| | | |
|---|---|---|
| ℞ | Sulphur Precip. | 15 parts |
| | Castor Oil | 50 " |
| | Cocoa Butter | 12 " |
| | Balsam Peru | 2 " |

Mix the sulphur and castor oil thoroughly, then add the cocoa butter by aid of gentle heat and finally the balsam. S. Rub the scalp morning and evening. (The hair should, of course, be cut short.)

### THE OLD OAKEN BUCKET.

(*Revised and Edited by a "Sanitarian."*)

With what anguish of mind I remember my childhood,
 Recalled in the light of a knowledge since gained;
The malarious farm, the wet, fungus-grown wild-wood;
 The chills then contracted that since have remained:
The scrum-covered duck pond, the pig-sty close by it,
 The ditch where the sour-smelling house dramage fell;
The damp, shaded dwelling, the foul barn-yard nigh it—
 But worse than all else was that terrible well;
And the old oaken bucket, the mould-crusted bucket,
 The moss-covered bucket that hung in the well.

Just think of it! *Moss* on the vessel that lifted
 The water I drank in the days called to mind,
Ere I knew what professors and scientists gifted
 In the water of wells by analysis find.
The rotting wood fibre, the oxide of iron,
 The algæ, the frog of unusual size,
The water, impure as the verses of Byron,
 Are things I remember with tears in my eyes.
And to tell the sad truth—though I shudder to think it—
 I considered that water uncommonly clear;
And often at noon, when I went there to drink it,
 I enjoyed it as much as I now enjoy beer.
How ardent I seized it with hands that were grimy,
 And quick to the mud-covered bottom it fell;
Then reeking with nitrates and nitrites, and slimy
 With matter organic, it rose from the well.
Oh! had I but realized, in time to avoid them,
 The dangers that lurked in that pestilent draught,
I'd have tested for organic germs and destroyed them
 With potassic permanganate ere I had quaffed;
Or, perchance, I'd have boiled it, and afterward strained it
 Through filters of charcoal and gravel combined;
Or, after distilling, condensed and regained it,

In potable form, with its filth left behind.
How little I knew of the dread typhoid fever
Which lnrked in the water I ventured to drink;
But since I've become a devoted believer
In the teachings of science I shudder to think.
And now, far removed from the scenes I'm describing,
The story for warning to others I tell,
As memory reverts to my youthful imbibing
And I gag at the thought of that horrible well,
And the old oaken bucket, the fungus-grown bucket—
In fact, the slop bucket—that hung in the well.

J. C. BAYLES.

*Brooklyu Medical Journal.*

---

### THE BIG MEETING.

### Railroad Rates to and from San Antonio.

Just as we go to press, we get the following, which is official:

International & Great Northern Railroad—no reduction.

Southern Pacific Railroad, one-sixth rebate.

San Antonio & Aransas Pass Railroad, one fare for the round trip.

Tickets will be on sale at all offices, and agents will be especially instructed as to these rates.

F. E. DANIEL,
Secretary.

---

DR. T. R. DICE, Utica, Mo., says: "I beg leave to state that I am well satisfied with the use of Crystalline Phosphate. I regard it as an improvement upon the liquid preparations in the market. Crystalline Phosphate is convenient to dispense, pleasant to the most fastidious taste, elegant in appearance, and decidedly in combination with nux vomica the best tonic I ever prescribed in atonic conditions of the stomach."

---

THE DIOS CHEMICAL CO., of St. Louis, have a whole page advertisement in this issue, to which attention is requested.

